# The roles of *BTG1* mRNA expression in cancers: A bioinformatics analysis

**DOI:** 10.3389/fgene.2022.1006636

**Published:** 2022-10-21

**Authors:** Hua-chuan Zheng, Hang Xue, Cong-yu Zhang, Kai-hang Shi, Rui Zhang

**Affiliations:** ^1^ Department of Oncology, The Affiliated Hospital of Chengde Medical University, Chengde, China; ^2^ Cancer Center, The First Affiliated Hospital of Jinzhou Medical University, Jinzhou, China; ^3^ Department of Dermatology, The Affiliated Hospital of Chengde Medical University, Chengde, China; ^4^ Department of Colorectal Surgery, Liaoning Cancer Hospital, Shenyang, China

**Keywords:** Btg1, bioinformatics analysis, carcinogenesis, aggressiveness, prognosis

## Abstract

BTG1 (B-cell translocation gene 1) may inhibit proliferation and cell cycle progression, induce differentiation, apoptosis, and anti-inflammatory activity. The goal of this study was to clarify the clinicopathological and prognostic significances of *BTG1* mRNA expression and related signal pathways in cancers. Using the Oncomine, TCGA (the cancer genome atlas), xiantao, UALCAN (The University of ALabama at Birmingham Cancer data analysis Portal), and Kaplan-Meier plotter databases, we undertook a bioinformatics study of *BTG1* mRNA expression in cancers. *BTG1* expression was lower in gastric, lung, breast and ovarian cancer than normal tissue due to its promoter methylation, which was the opposite to *BTG1* expression. *BTG1* expression was positively correlated with dedifferentiation and histological grading of gastric cancer (*p* < 0.05), with squamous subtype and young age of lung cancer (*p* < 0.05), with infrequent lymph node metastasis, low TNM staging, young age, white race, infiltrative lobular subtype, Her2 negativity, favorable molecular subtyping, and no postmenopause status of breast cancer (*p* < 0.05), and with elder age, venous invasion, lymphatic invasion, and clinicopathological staging of ovarian cancer (*p* < 0.05). *BTG1* expression was negatively correlated with favorable prognosis of gastric, lung or ovarian cancer patients, but the converse was true for breast cancer (*p* < 0.05). KEGG (Kyoto Encyclopedia of Genes and Genomes) analysis showed that the top signal pathways included cytokine-cytokine receptor interaction, cell adhesion molecules, chemokine, immune cell receptor and NF (nuclear factor)-κB signal pathways in gastric and breast cancer. The top hub genes mainly contained CD (cluster of differentiation) antigens in gastric cancer, FGF (fibroblast growth factor)-FGFR (FGF receptor) in lung cancer, NADH (nicotinamide adenine dinucleotide): ubiquinone oxidoreductase in breast cancer, and ribosomal proteins in ovarian cancer. *BTG1* expression might be employed as a potential marker to indicate carcinogenesis and subsequent progression, even prognosis.

## Introduction

BTG1 (B-cell translocation gene 1) is reported to suppress cell proliferation and cell cycle progression, and induce cell differentiation due to its interaction with the myogenic factor MyoD (Busson et al., 2005), protein arginine methyltransferase 1 (Lin et al., 1996), and human carbon catabolite repressor protein-associative factor 1 (Bogdan et al., 1998). BTG1 has also been demonstrated to promote Hoxb9-induced transcription to suppress proliferation in HeLa cells (Prévôt et al., 2000). Additionally, BTG1 mediates the apoptotic induction, as evidenced by BTG1 overexpression in apoptotic cells (Corjay et al., 1998) and the contribution of BTG1 to anti-sense Bcl-2- induced cytotoxicity (Nahta et al., 2006). Liu et al. (2015) found that BTG1 potentiated apoptosis and suppressed proliferation in renal clear cell carcinoma by interacting with PRMT1. BTG1 could reverse the miR-22-induced inhibition of autophagy (Zhang et al., 2015), while miR-4295 significantly promoted proliferation, colony formation, and migration of bladder cancer cell *via* directly targeting BTG1 (Nan et al., 2016). By inhibiting BTG1, miR-511 strengthened the proliferation of human hepatoma cells, while miR-301A promoted the development of colitis-associated cancer (He et al., 2017; Zhang et al., 2017). BTG1 functioned as a direct target of miR-330-3p, and miR-27a-3p in hepatocellular carcinoma and ovarian cancer cells, thereby weakened cell viability, migration and invasion, and promoted cell apoptosis (Li et al., 2019; Zhao et al., 2019). BTG1 was shown to prevent antigen from inducing molecular features of *in vitro* allergic reactions as a direct target of miR-183-5p (Kim et al., 2020). Ni et al. (2021) found that miR-141-5p enhanced the proliferation and inhibited apoptosis by targeting *BTG1* in cervical Cancer cells. Cheng et al. (2021) revealed that the exosomal miR-301a-3p promotes the proliferation and invasion of nasopharyngeal carcinoma squamous cells by targeting *BTG1*.

Su et al. (2019) reported that BTG1 overexpression triggered G_1_/S phase cell cycle arrest and increased apoptosis in HCT-116 cells *via* the ERK (extracellular regulated protein kinases)/MEK (map kinase kinases) signaling pathway. Zhu et al. (2015) showed that BTG1 enhanced the radiation sensitivity of human breast cancer by inducing cell cycle arrest, the formation of reactive oxygen species, chromosomal aberrations and apoptosis *via* inhibition of the PI3K/Akt signaling pathway. BTG1 overexpression was also found to suppress proliferation, tumor growth and lung metastasis, induce differentiation, autophagy, and apoptosis, and mediate chemosensitivity in colorectal or gastric cancer cells (Zheng et al., 2015; Zhao et al., 2017). Xue et al. (2021) found that chidamide triggered BTG1-mediated autophagy and reverses the chemotherapy resistance in relapsed/refractory B-cell lymphoma. However, BTG1 overexpression was found to promote invasion and metastasis of colorectal cancer in our previous study (Zhu et al., 2014; Szász et al., 2016; Zhao et al., 2020).

In the present study, we aimed to clarify the clinicopathological and prognostic significances of *BTG1* mRNA expression in cancers by bioinformatics analysis of high-throughput cDNA array and RNA sequencing using online Oncomine, TCGA(the cancer genome atlas), xiantao, UALCAN (The University of ALabama at Birmingham Cancer data analysis Portal) and Kaplan-Meier plotter. According to the cancer types of Kaplan-Meier plotter (before 2018), we chose gastric, lung, breast and ovarian cancers for *BTG1* analysis.

## Methods

### Oncomine database analysis

The individual gene expression level of *BTG1* mRNA was analyzed using Oncomine (www.oncomine.org), a cancer microarray database and a web-based data mining platform for a new discovery from genome-wide expression analyses. We compared the differences in *BTG1* mRNA levels between normal tissue and cancer. All data were log-transformed, with median centered per array centered and standard deviation normalized to each array.

### TCGA database analysis

The Cancer Genome Atlas (TCGA, https://cancergenome.nih.gov/) database was used to download expression data (RNA-seqV2) and clinicopathological data of gastric (*n* = 392), lung (*n* = 865), breast (*n* = 1,093), and ovarian (*n* = 304) cancer patients. We integrated the raw data, analyzed *BTG1* expression in the cancers, and compared it with clinicopathological and prognostic data from the cancer patients. A student t test was used to compare the means. Kaplan-Meier survival plots were generated with survival curves compared by log-rank statistic. Cox’s proportional hazards model was employed for multivariate analysis. Two-sided *p* < 0.05 was considered statistically significant. SPSS 17.0 software was employed to analyze all the data.

### GEO analysis

The mRNA expression profile of GSE38666 (platform: Affymetrix-GPL570) and GSE26712 (platform: Affymetrix-GPL96) was obtained from NCBI GEO database (https://www.ncbi.nlm.nih.gov/geo/), and R was used for the analysis of the *BTG1* mRNA expression between ovarian cancer and normal tissues.

### Kaplan-Meier plotter analysis

The prognostic significance of *BTG1* mRNA in gastric, lung, breast and ovarian cancers was also analyzed by the Kaplan-Meier plotter. (http://kmplot.com).

### UALCAN analysis

The expression and methylation of *BTG1* gene and the relationship between BTG1 expression and immune cells infiltration were analyzed using UALCAN database (http://ualcan.path.uab.edu). They were also compared to clinicopathologic and prognostic parameters associated with gastric, lung, breast, and ovarian cancers.

### Xiantao analysis

The expression and methylation of *BTG1* gene and the relationship between BTG1 expression and immune cells infiltration were analyzed using xiantao platform (https://www.xiantao.love/).

Additionally, we found the differential and related genes using xiantao. The PPI (protein-protein interaction) network was built using the differential genes, and the important hub genes were identified. These genes were submitted to KEGG (Kyoto Encyclopedia of Genes and Genomes) analysis in order to build signal pathways.

## Results

### The clinicopathological and prognostic significances of *BTG1* mRNA expression in gastric cancer

**FIGURE 1:**
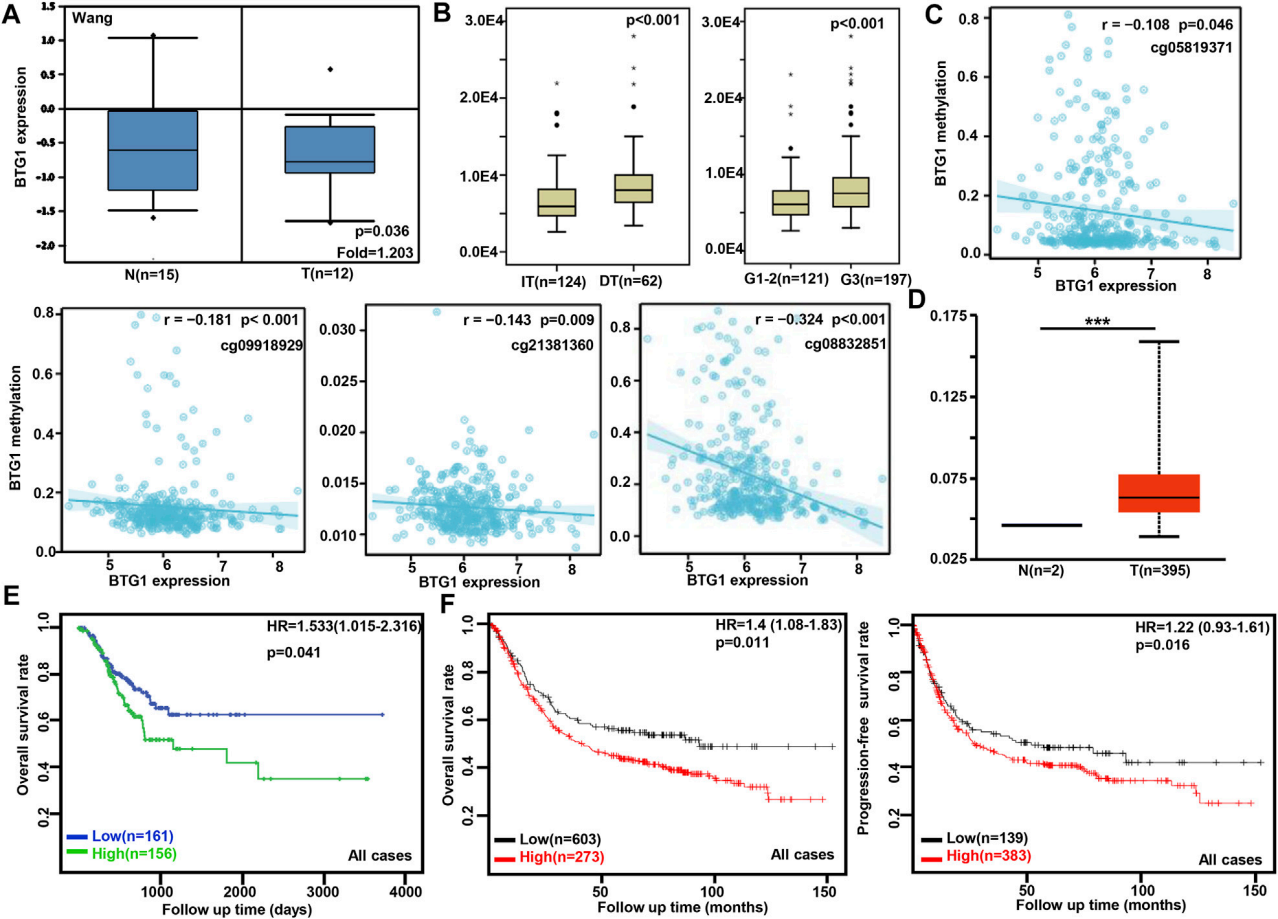
The clinicopathological and prognostic significances of *BTG1* mRNA expression in gastric cancer. Wang’s **(A)** dataset was used for bioinformatics analysis to explore *BTG1* expression in gastric cancer. A lower *BTG1* expression was detectable in gastric cancer than that in normal mucosa (*p* < 0.05). TCGA database showed that *BTG1* was negatively correlated with Lauren’s classification and histological staging of gastric cancer **(B)** *p* < 0.05. The negative relationship between *BTG1* expression and methylation was analyzed in gastric cancer using xiantao database **(C)**. Its methylation was higher in gastric cancer than normal tissues in UALCAN **(D)**. *BTG1* expression was negatively correlated with overall survival rate of the cancer patients by TCGA **(E)** *p* < 0.05. It was the same for the overall and progression-free survival rates of the patients with gastric cancer according to the data from Kaplan-Meier plotter **(F)** *p* < 0.05. Note: N, normal tissue; T, tumor; HR, hazard ratio; IT, intestinal-type; DT, diffuse-type.

According to Wang’s database, we found that *BTG1* mRNA expression was lower in gastric cancer than in normal tissues (Figure 1A, *p* < 0.05). In TCGA data, *BTG1* expression was positively correlated with dedifferentiation and histological grading of gastric cancer (Figure 1B, *p* < 0.05). Using the xiantao tool, we discovered a negative correlation between *BTG1* mRNA and its methylations (cg05819371, cg09918929, cg21381360, and cg08832851) (Figure 1C, *p* < 0.05). The UALCAN showed that *BTG1* methylation was higher in gastric cancer than in normal tissues (Figure 1D, *p* < 0.05). According to the TCGA data, *BTG1* mRNA expression was negatively related to the overall survival of the gastric cancer patients (Figure 1E, *p* < 0.05). By the Kaplan-Meier plotter, a higher *BTG1* expression was negatively correlated with overall and progression-free survival rates of all cancer patients, male or perforating cancer patients and patients receiving 5-FU-based adjuvant (Figure 1F; Table 1, *p* < 0.05). As shown in Table 1, negative correlation between overall survival and *BTG1* expression was found in the patients with stage II and IV, T2, N3, intestinal and mixed or Her2-positive cancers (*p* < 0.05). It was similar for progression-free survival in female, male, or poorly-differentiated cancer patients (*p* < 0.05). Negative association between *BTG1* expression and overall prognosis was observed in the cancer patients only receiving surgical operation (Table 1, *p* < 0.05).

**TABLE 1:** The prognostic significance of *BTG1* mRNA in gastric cancer.

Clinicopathological features	Overall survival		Progression-free survival	
	Hazard ratio	*p*-value	Hazard ratio	*p*-value
Sex				
Female	0.69 (0.45–1.07)	0.098	0.65 (0.43–0.99)	0.045
Male	1.65 (1.22–2.24)	0.001	1.45 (1.08–1.94)	0.012
T				
2	1.65 (1.08–2.53)	0.019	1.44 (0.9–2.32)	0.13
3	0.75 (0.51–1.08)	0.12	—	—
4	0.54 (0.2–1.48)	0.23	—	—
N				
0	1.98 (0.84–4.67)	0.11	1.68 (0.72–3.91)	0.22
1–3	1.29 (0.99–1.69)	0.062	1.24 (0.92–1.67)	0.16
1	1.49 (0.98–2.27)	0.063	1.33 (0.89–1.98)	0.16
2	0.72 (0.46–1.13)	0.15	0.79 (0.51–1.21)	0.28
3	2.07 (1.16–3.71)	0.013	1.71 (0.97–3.01)	0.061
M				
0	—	—	1.28 (0.94–1.75)	0.12
1	1.37 (0.99–1.91)	0.058	1.63 (0.85–3.13)	0.14
TNM staging				
I	1.67 (0.54–5.18)	0.37	0.5 (0.16–1.57)	0.23
II	2.5 (1.05–5.97)	0.032	1.89 (0.84–4.26)	0.12
III	0.81 (0.56–1.19)	0.28	0.74 (0.48–1.13)	0.16
IV	1.73 (1.14–2.65)	0.0099	1.38 (0.92–2.08)	0.11
Differentiation				
Well-differentiated	—	—	—	—
Moderately-differentiated	0.62 (0.32–1.21)	0.16	0.66 (0.35–1.24)	0.2
Poorly-differentiated	0.61 (0.37–1.01)	0.05	0.59 (0.37–0.94)	0.026
Lauren’s classification				
Intestinal-type	1.49 (1.03–2.13)	0.031	1.38 (0.95–2.02)	0.092
Diffuse-type	1.18 (0.82–1.68)	0.38	0.81 (0.56–1.18)	0.27
Mixed-type	3.52 (1.11–11.15)	0.023	0.5 (0.18–1.35)	0.16
Her2 positivity				
−	1.27 (0.97–1.66)	0.078	1.23 (0.91–1.65)	0.18
+	1.73 (1.11–2.71)	0.015	1.42 (0.89–2.27)	0.14
Perforation				
−	0.57 (0.38–0.86)	0.0059	0.61 (0.42–0.9)	0.011
Treatment				
Surgery alone	1.56 (1.13–2.14)	0.0063	1.31 (0.96–1.77)	0.083
5-FU-based adjuvant	0.21 (0.06–0.71)	0.0058	0.16 (0.05–0.55)	0.00095
Other adjuvant	0.6 (0.25–1.47)	0.26	1.54 (0.69–3.44)	0.29

### The clinicopathological and prognostic significances of *BTG1* mRNA expression in lung cancer

**FIGURE 2:**
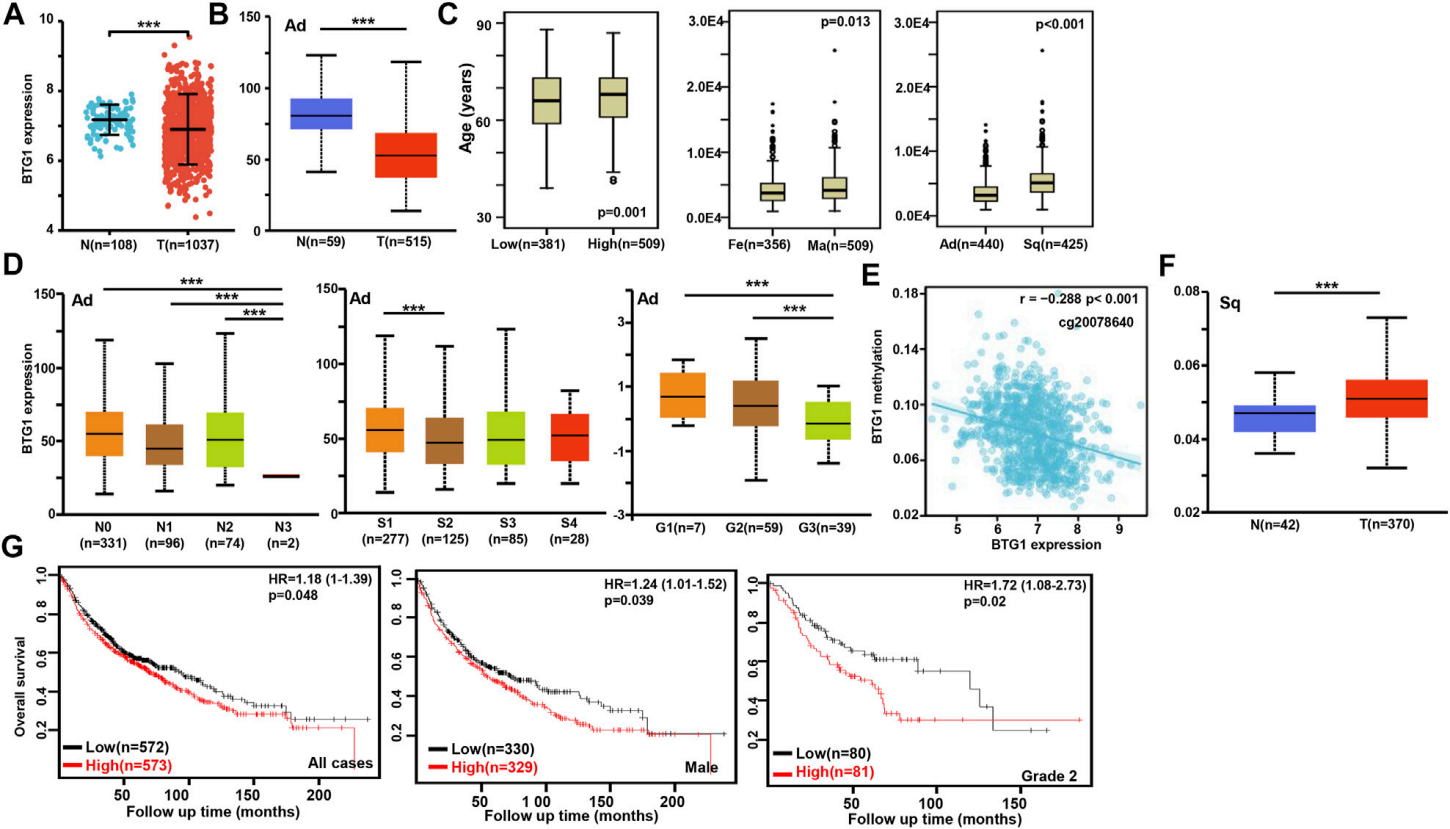
The clinicopathological and prognostic significances of *BTG1* mRNA expression in lung cancer. Xiantao **(A)** and UALCAN **(B)** datasets were employed for bioinformatics analysis to analyze BTG1 expression during lung carcinogenesis. *BTG1* expression was downregulated in lung cancer, compared with normal tissue (*p* < 0.05). *BTG1* expression was compared with age, gender and histological subtyping of the cancer patients by TCGA **(C)**, and with N staging, clinicopathological staging and histological grading by UALCAN **(D)**. The negative relationship between *BTG1* expression and methylation was analyzed in lung cancer using xiantao database **(E)**. Its methylation was higher in lung cancer than normal tissues in UALCAN **(F)**. The correlation between *BTG1* expression and overall or post-progression survival rate of the patients with lung cancer was analysis using Kaplan-Meier plotter **(G)**. Note: Ad, adenocarcinoma; Sq, squamous cell carcinoma; Fe, female; Ma, male; S, stage; HR, hazard ratio.

According to xiantao (Figure 2A) and UALCAN databases (Figure 2B), *BTG1* expression was lower in lung cancer than in normal tissue *p* < 0.05). In TCGA data, *BTG1* expression was higher in squamous cell carcinoma than adenocarcinoma, in male than female cancer patients, and in elder than younger cancer patients (Figure 2C, *p* < 0.05). In UALCAN data, *BTG1* expression was lower in N_3_ than N_0_, N_1_ and N_2_, S_2_ than S_1_, and G_3_ than G_1_ and G_2_ cancer (Figure 2D, *p* < 0.05). There was a negative correlation between *BTG1* mRNA and methylation (cg20078640, cg05819371, cg09918929, cg06551025, cg13132650, cg04211745, cg04100724, cg21381360, cg25218905 and cg08832851) by xiantao (Figure 2E, *p* < 0.05). *BTG1* methylation was higher in lung cancer than in normal tissues by UALCAN (Figure 2F, *p* < 0.05). According to Kaplan-Meier plotter, we found that the higher *BTG1* expression was negatively correlated with the overall rate of all, male or G_2_ cancer patients (Figure 2G, *p* < 0.05). The Cox’s risk proportional analysis showed that younger age, lymph node metastasis, TNM staging and *BTG1* hypoexpression were independent factors for worse prognosis of the patients with lung cancer (Table 2, *p* < 0.05).

**TABLE 2:** Multivariate analysis of hazard factors of the prognosis of the patients with lung cancer.

Clinicopathological features	Hazard ratio (95% CI)	*p*-value
Age (<60/≧60 years)	2.175 (1.467–3.226)	<0.001
Gender (Female/male)	0.589 (0.081–4.266)	0.600
T stage (T_1-2_/T_3-4_)	0.713 (0.401–1.269)	0.250
Lymph node metastasis (−/+)	1.761 (1.066–2.911)	0.027
TNM staging (I-II/III-IV)	2.341 (1.343–4.083)	0.003
Histological classification (Ad/Sq)	1.090 (0.650–1.828)	0.744
*BTG1* mRNA expression (low/high)	0.503 (0.329–0.770)	0.002

Ad, adenocarcinoma; Sq, squamous cell carcinoma; CI, confidence interval.

### The clinicopathological and prognostic significances of *BTG1* mRNA expression in breast cancer

**FIGURE 3:**
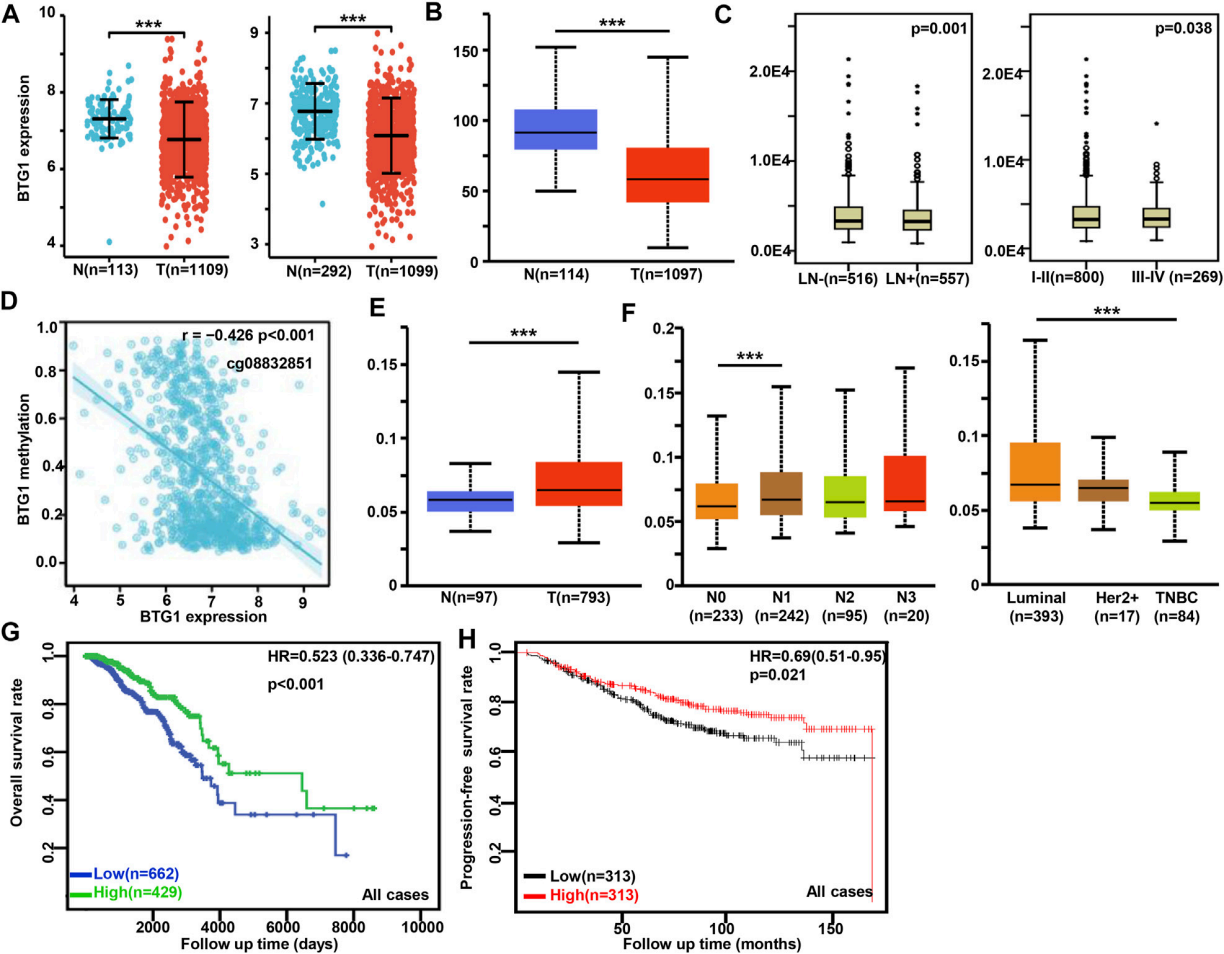
The clinicopathological and prognostic significances of *BTG1* mRNA expression in breast cancer. Xiantao **(A)** and UALCAN **(B)** dataset was used for bioinformatics analysis to explore *BTG1* expression in breast cancer. A lower *BTG1* expression was detectable in breast cancer than that in normal mucosa (*p* < 0.05). TCGA database showed that *BTG1* was negatively correlated with lymph node metastasis (LN) and clinicopathological staging of breast cancer **(C)** *p* < 0.05. The negative relationship between *BTG1* expression and methylation was analyzed in breast cancer using xiantao database **(D)**. Its methylation was higher in breast cancer than normal tissues in UALCAN **(E)**, and compared with N staging and molecular subtyping of breast cancer **(F)**. *BTG1* expression was positively correlated with overall survival rate of the cancer patients by TCGA **(G)** *p* < 0.05 and progression-free survival by Kaplan-Meier plotter **(H)** *p* < 0.05. Note: N, normal tissue; T, tumor; TNBC, triple-negative breast cancer; HR, hazard ratio.

*BTG1* was more expressed in breast normal tissue than in cancer according to xiantao (Figure 3A) and UALCAN databases (Figure 3B, *p* < 0.05). TCGA database showed that *BTG1* expression was negatively associated with lymph node metastasis and TNM staging of breast cancer (Figure 3C, *p* < 0.05). There was a negative correlation between *BTG1* mRNA and methylation (cg05819371, cg09918929, cg21381360, and cg08832851) by xiantao (Figure 3D, *p* < 0.05). *BTG1* methylation was higher in breast cancer than normal tissues (Figure 3E, *p* < 0.05), N1 than N0, and Luminal than triple-negative breast cancer (TNBC) patients (Figure 3F, *p* < 0.05) by UALCAN. As summarized in Table 3, *BTG1* mRNA expression was positively correlated with young patients, white race, infiltrative lobular carcinoma, Her2 negativity, better molecular subtyping, and no postmenopause status (*p* < 0.05). TCGA data showed that *BTG1* expression was positively associated with a high overall survival rate of breast cancer patients (Figure 3G *p* < 0.05). According to Kaplan-Meier plotter, a higher *BTG1* expression was positively correlated with overall survival rates of all or luminal-B cancer patients (Figure 3H, *p* < 0.05). Patients with Her2-negative and luminal-B cancer who had high *BTG1* expression had a longer time without distant metastasis than those who had low *BTG1* expression (*p* < 0.05, data not shown). There appeared to be a positive relationship between *BTG1* expression and the progression-free survival rate of cancer patients without chemotherapy or margin invasion (*p* < 0.05, data not shown). The overall survival rate of the patient with ER (estrogen receptor)-negative, grade-3, or luminal-B cancer was higher in the group of high *BTG1* expression than that in its low expression (*p* < 0.05, data not shown).

**TABLE 3:** The correlation of *BTG1* mRNA expression with clinicopathological features of breast cancer.

Characteristic	Variables	Low expression	High expression	*p* -value
Age (years), n (%)	≦60	281 (25.9%)	320 (29.5%)	0.022
	>60	260 (24%)	222 (20.5%)	
Race, n (%)	Asian	34 (3.4%)	26 (2.6%)	0.021
	Black or African American	104 (10.5%)	77 (7.7%)	
	White	354 (35.6%)	399 (40.1%)	
Histological type, n (%)	IDC	414 (42.4%)	358 (36.6%)	<0.001
	I LC	66 (6.8%)	139 (14.2%)	
Her2 status, n (%)	Negative	260 (35.8%)	298 (41%)	0.003
	Indeterminate	8 (1.1%)	4 (0.6%)	
	Positive	96 (13.2%)	61 (8.4%)	
PAM50, n (%)	Normal	9 (0.8%)	31 (2.9%)	<0.001
	Luminal A	263 (24.3%)	299 (27.6%)	
	Luminal B	123 (11.4%)	81 (7.5%)	
	Her2+	47 (4.3%)	35 (3.2%)	
	Basal-like	99 (9.1%)	96 (8.9%)	
Menopause status, n (%)	Pre	102 (10.5%)	127 (13.1%)	0.043
	Peri	17 (1.7%)	23 (2.4%)	
	Post	374 (38.5%)	329 (33.8%)	

IDC, infiltrating ductal carcinoma; ILC, infiltrating lobular carcinoma.

### The clinicopathological and prognostic significances of *BTG1* mRNA expression in ovarian cancer

**FIGURE 4:**
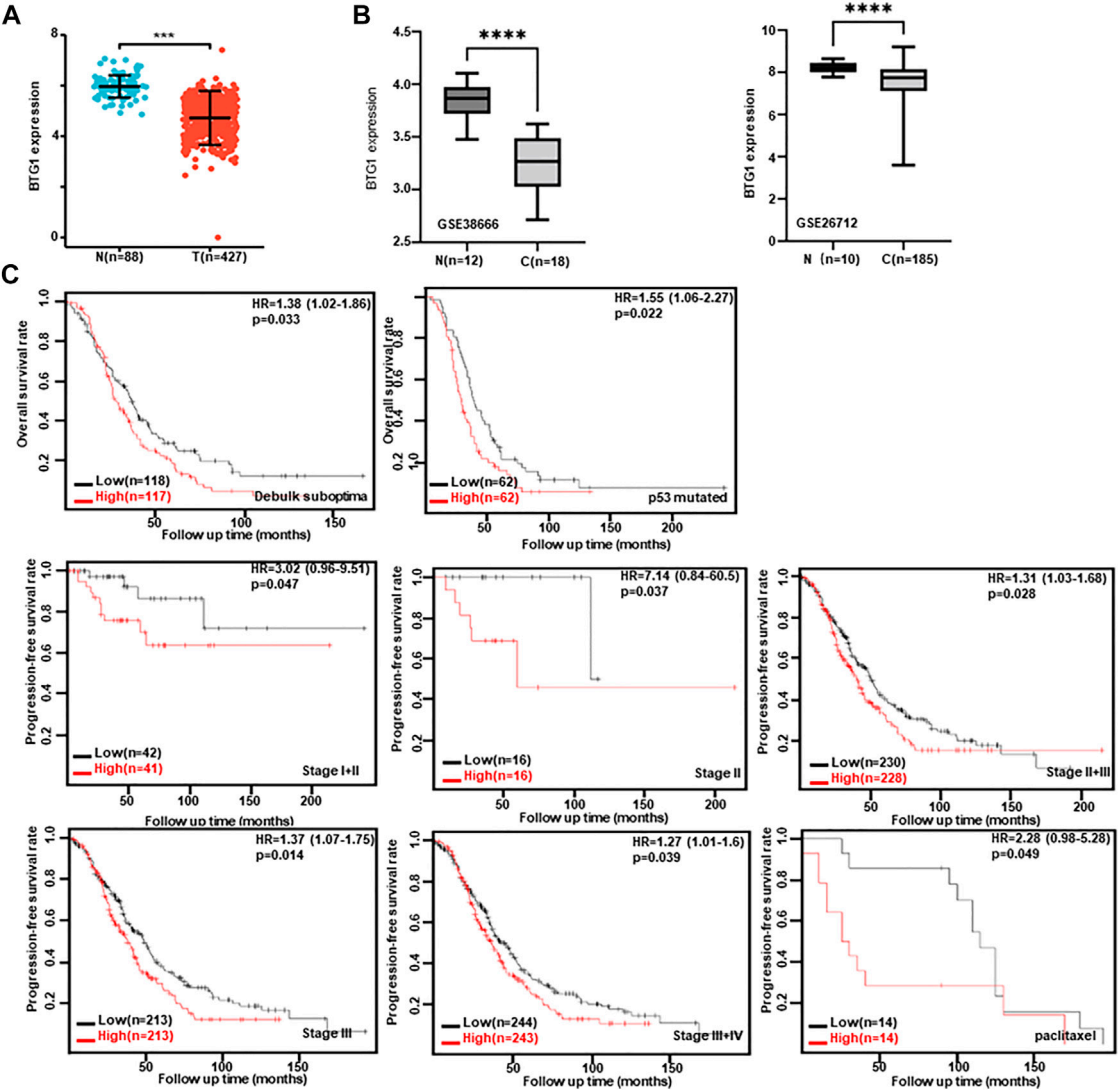
The clinicopathological and prognostic significances of *BTG1* mRNA expression in ovarian cancer. Both xiantao **(A)** and GEO **(B)** databases were employed for bioinformatics analysis to observe *BTG1* expression in ovarian cancer. A lower *BTG1* expression was detectable in ovarian carcinoma than normal tissue (*p* < 0.05). The correlation between *BTG1* expression and overall, or post-progression survival rate of the patients with ovarian cancer, even stratified by different clinicopathological parameters **(C)** *p* < 0.05. HR, hazard ratio.

Then, we used xiantao database to perform bioinformatics analysis and found that *BTG1* expression was lower in ovarian cancers than normal tissues (Figure 4A, *p* < 0.05), in line with GEO data (GSE38666 and GSE26712, Figure 4B). As shown in Table 4, *BTG1* expression was positively associated with elder age, venous invasion, lymphatic invasion, and FIGO (International Federation of Gynecology and Obstetrics) staging of ovarian cancer (*p* < 0.05). According to Kaplan-Meier plotter, a higher *BTG1* expression was negatively correlated with the overall survival rates of Dubulk suboptimal and p53-mutant cancer patients (Figure 4C, *p* < 0.05). Stage I + II, II, II + III, III, and III + IV cancer patients with high *BTG1* expression showed a short progression-free survival time than those with its low expression (Figure 4B, *p* < 0.05). There appeared a negative relationship between *BTG1* expression and the overall survival rate of the cancer patients with paclitaxel treatment (Figure 4B, *p* < 0.05).

**TABLE 4:** The correlation of *BTG1* mRNA expression with clinicopathological features of ovarian cancer.

Characteristic	Variables	Low expression	High expression	*p*-value
Age (years), n (%)	≦60	112 (29.6%)	96 (25.3%)	0.108
	>60	77 (20.3%)	94 (24.8%)	
Venous invasion, n (%)	No	21 (20%)	20 (19%)	0.480
	Yes	27 (25.7%)	37 (35.2%)	
Lymphatic invasion, n (%)	No	30 (20.1%)	18 (12.1%)	0.036
	Yes	43 (28.9%)	58 (38.9%)	
FIGO stage, n (%)	Stage 1	0 (0%)	1 (0.3%)	0.022
	Stage 2	12 (3.2%)	11 (2.9%)	
	Stage 3	138 (36.7%)	157 (41.8%)	
	Stage 4	38 (10.1%)	19 (5.1%)	

FIGO, federation of gynecology and obstetrics.

### The relationship between *BTG1* mRNA expression and the infiltrating immune cells in cancers

**FIGURE 5:**
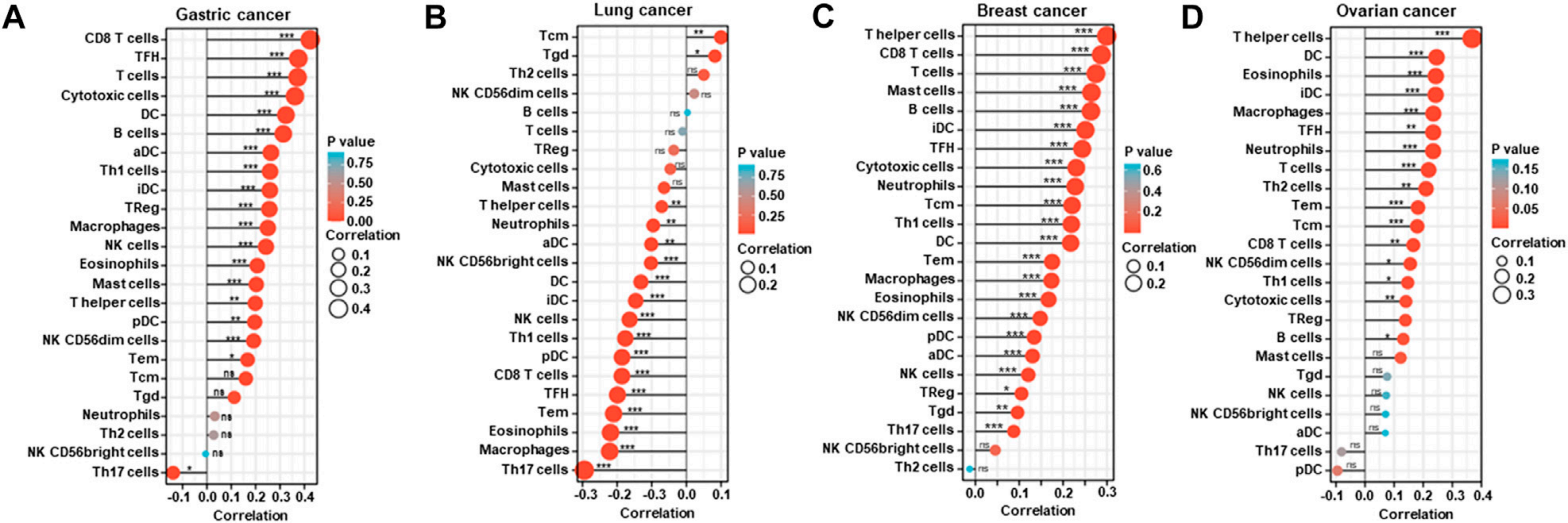
The relationship between *BTG1* mRNA expression and immune infiltration in cancers. The enrichment of immune cells was explored between low and high expression of *BTG1* in gastric **(A)**, lung **(B)**, breast **(C)**, and ovarian **(D)** cancers using xiantao. ns, not significant; *p < 0.05; **p < 0.01; ***p < 0.001.

According to xiantao, *BTG1* mRNA expression was positively related to the infiltration of CD8^+^ T cells, TFH, T cells, cytotoxic cells, DC, B cells, aDC, Th1 cells, iDC, TReg, macrophages, NK cells, eosinophils, mast cells and T helper cells, pDC, NK CD56dim cells and Tem, but negatively to Th17 cells in gastric cancer (Figure 5A, *p* < 0.05). It was positively related to the infiltration of Tcm and Tgd, but negatively to T helper cells, neutrophils, aDC, NK CD56bright cells, DC, iDC, NK cells, Th1 cells, pDC, CD8^+^ T cells, TFH, Tem, eosinophils, macrophages and Th17 cells in lung cancer (Figure 5B, *p* < 0.05). It was positively related to the infiltration of T helper cells, CD8^+^ T cells, T cells, mast cells, B cells, iDC, TFH, cytotoxic cells, neutrophils, Tcm, Th1 cells, DC, Tem, macrophages, eosinophils, NK CD56bright cells, pDC, aDC, NK cells, TReg, Tgd and Th17 cells in breast cancer (Figure 5C, *p* < 0.05). It was positively related to the infiltration of T helper cells, DC, eosinophils, iDC, macrophages, TFH, neutrophils, T cells, Th2 cells, Tem, Tcm, CD8^+^ T cells, NK CD56dim cells, Th1 cells, cytotoxic cells and B cells in ovarian cancer (Figure 5D, *p* < 0.05).

### The *BTG1*-related genes and pathways in cancers

**FIGURE 6:**
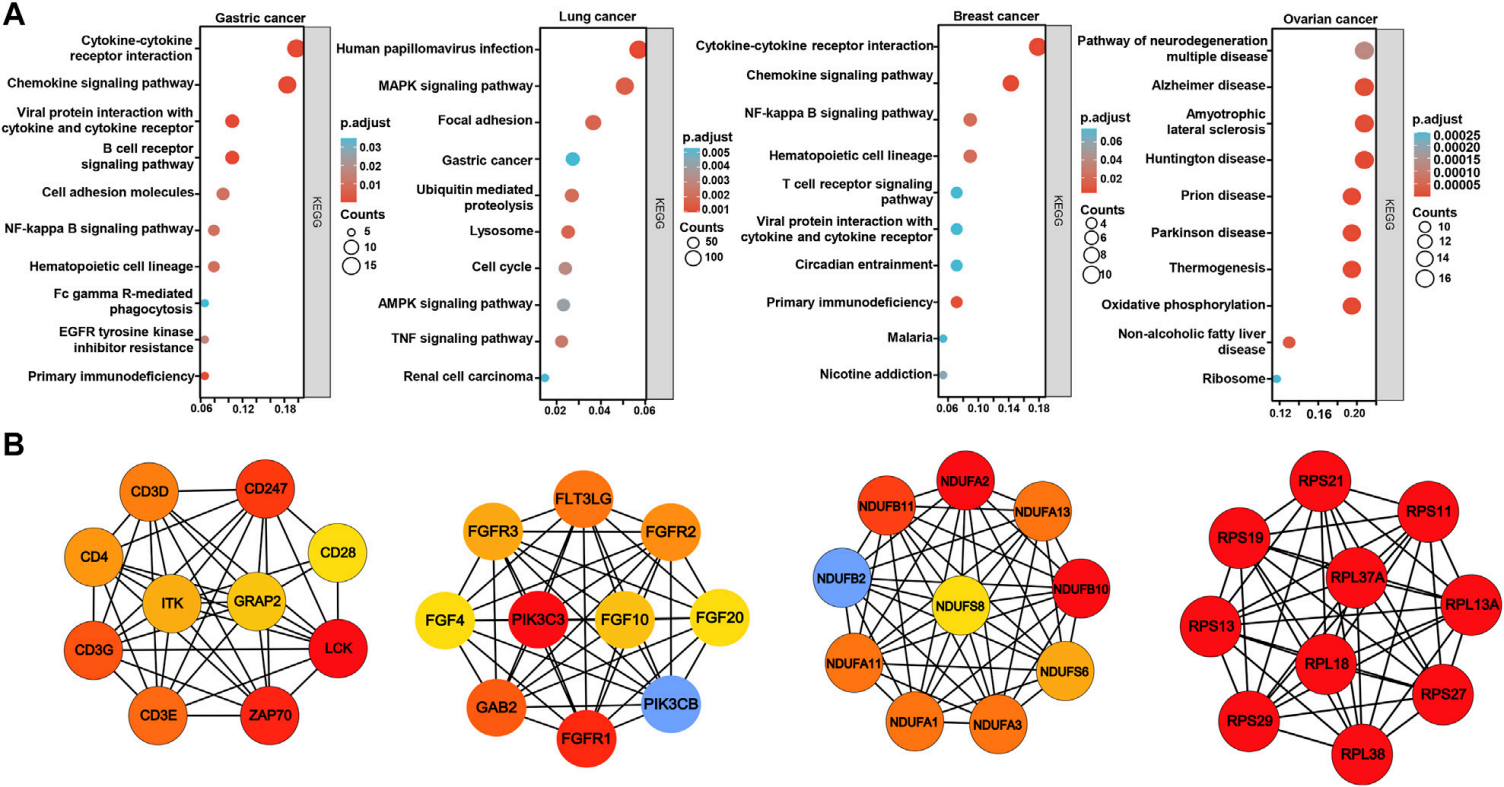
The differential genes and related signal pathways about *BTG1* in cancers The differential genes of *BTG1* were subjected to the signal pathway analysis using KEGG **(A)**. STRING was used to identify the protein-protein interaction network of differential genes about *BTG1* in cancers, and cytoscape was employed to find out the top 10 hub nodes ranked by degree **(B)**.

On the xiantao platform, we discovered the genes that differed between low and high expression groups of *BTG1* mRNA in cancers. KEGG analysis showed that the top signal pathways included cytokine-cytokine receptor interaction, cell adhesion molecules, chemokine, B cell receptor and NF(nuclear factor)-κB signal pathways in gastric cancer, focal adhesion, lysosome, cell cycle, MAPK (mitogen-activated protein kinase), AMPK (Adenosine 5’-monophosphate-activated protein kinase) and TNF (tumor necrosis factor) signal pathways in lung cancer, cytokine-cytokine receptor interaction, chemokine, T cell and NF-κB signal pathways in breast cancer, and neural diseases, oxidative phosphorylation, non-alcoholic fatty liver disease and ribosome in ovarian cancer (Figure 6A). In addition, the STRING was used to identify the PPI pairs and the cytoscape to find out the top 10 nodes ranked by degree (Figure 6B). The top hub genes mainly contained CD (cluster of differentiation) antigens in gastric cancer, FGF (fibroblast growth factor)-FGFR (FGF receptor) and downstream genes in lung cancer, mitochondrial NADH (nicotinamide adenine dinucleotide): ubiquinone oxidoreductase in breast cancer and ribosomal proteins in ovarian cancer.

**FIGURE 7:**
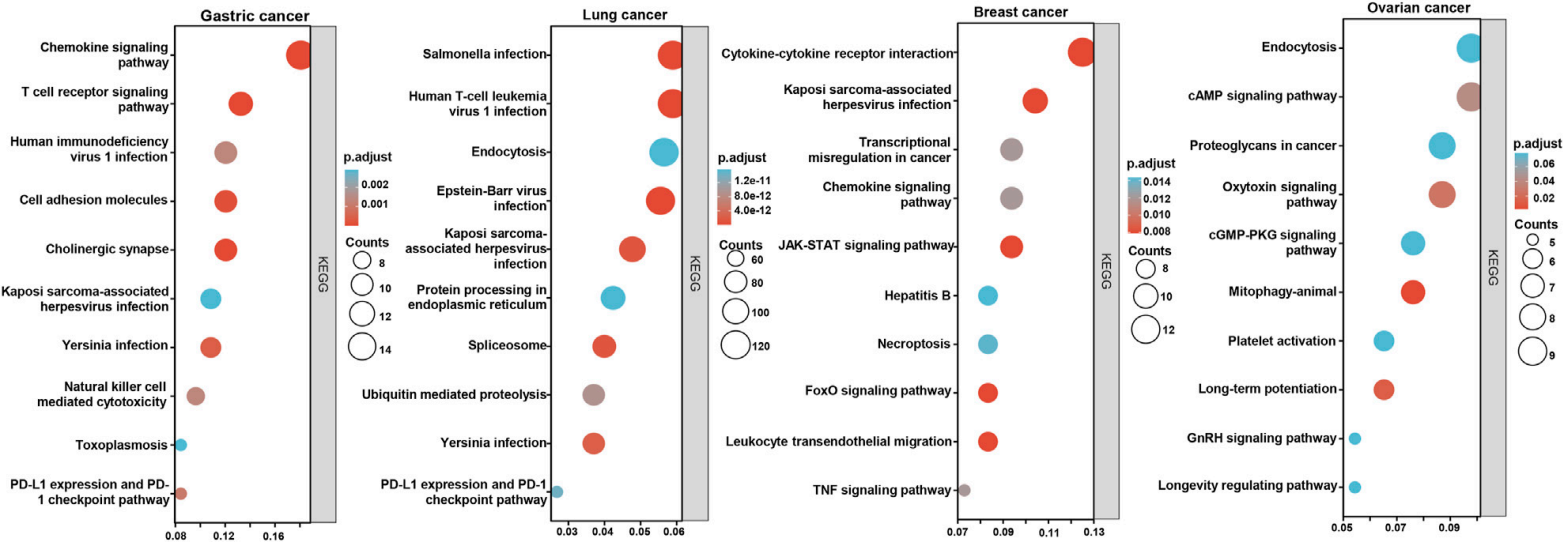
The *BTG1*-related signal pathways in cancers. The top *BTG1*-related genes were screened and classified into the signal pathways of KEGG using xiantao database.

According to the xiantao database, the *BTG1*-correlated genes in cancers were analyzed and subjected to the KEGG analysis (Figure 7). The *BTG1*-correlated genes were involved in cell adhesion, chemokine, T cell receptor and PD-L1 signal pathways for gastric cancer, endocytosis, infection-related diseases, and spliceosome, ubiquitin-mediated proteolysis and PD-L1 signal pathway for lung cancer, cytokine-cytokine receptor interaction, chemokine, JAK-STAT, FoxO and TNF signal pathway for breast cancer, endocytosis, cAMP, oxytoxin, cGMP and GnRH signal pathways for ovarian cancer.

## Discussion

Overexpression of BTG1 inhibited the proliferation, migration and invasion of hepatocytes, thyroid, nasopharynx, esophagus, breast and non-small cell lung cancer cells and induced apoptosis and cell cycle arrest by downregulating the expression of Cyclin D1, Bcl-2 and MMP-9 (Sun et al., 2014a; Sun et al., 2014b; Sun et al., 2014c; Sun et al., 2014d; Lu et al., 2014; Sheng et al., 2014). In ovarian cancer, BTG1 expression caused lower growth rate, high cisplatin sensitivity, G_1_ arrest, apoptosis, and decreased migration and invasion by down-regulating the expression of PI3K (phosphatidylinositol 3-kinase), PKB (protein kinase B), Bcl-xL, survivin, VEGF (vascular epithelial growth factor), and MMP (matrix metalloproteinase)-2 (Zhao et al., 2013). The chemosensitivity of BTG1 transfectants to paclitaxel, cisplatin, MG132 (proteasome inhibitor) or SAHA (histone deacetylase inhibitor) was positively correlated with its apoptotic induction of colorectal cancer cells (Zhao et al., 2017). BTG1 overexpression suppressed tumor growth and lung metastasis of gastric and colorectal cancer cells by inhibiting proliferation, and enhancing autophagy and apoptosis in xenograft models (Zheng et al., 2015; Zhao et al., 2017). Taken together, BTG1 should be used as a molecular target for cancer gene therapy.

Jung et al. (2018) found that *BTG1* expression was lower in colorectal cancer than in control, and in metastatic cancer than in primary cancer, due to the hypermethylation of the *BTG1* promoter. BTG1 expression was decreased in hepatocellular, thyroid, nasopharyngeal, esophageal, breast, colorectal, and non-small cell lung cancers, and negatively associated with aggressive behaviors (Sun et al., 2014a; Sun et al., 2014b; Sun et al., 2014c; Sun et al., 2014d; Lu et al., 2014; Sheng et al., 2014; Zhu et al., 2014). Decreased BTG1 expression in gastric cancer was positively correlated with depth of invasion, lymphatic and venous invasion, lymph node metastasis, TNM staging and worse prognosis (Zhu et al., 2015), but the lower BTG1 expression in ovarian cancer was positively correlated with FIGO staging (Sheng et al., 2014). Reportedly, the downregulated *BTG1* expression is positively correlated with its promoter methylation in colorectal, gastric and ovarian cancers (Kanda et al., 2015; Zheng et al., 2015; Kim et al., 2017). Here, we found that *BTG1* expression was lower in gastric, lung, breast and ovarian cancer than normal tissue due to its promoter methylation, which was opposite of *BTG1* expression. Moreover, *BTG1* expression was positively correlated with dedifferentiation and histological grading of gastric cancer, and venous invasion, lymphatic invasion, and TNM staging of ovarian cancer, but negatively associated with lymph node metastasis and TNM staging of breast cancer. In breast cancer, the relation of *BTG1* expression to TNM staging was the opposite to that between *BTG1* methylation and TNM staging. *BTG*1 expression was higher in pulmonary squamous cell carcinoma than in adenocarcinoma, which provided another evidence that squamous cell carcinoma but not adenocarcinoma showed higher *BTG1* expression than normal tissue. These findings suggested that aberrant *BTG1* expression was positively correlated with carcinogenesis, histogenesis and subsequent progression because of its aberrant promoter methylation. The contradictory data in our and other studies could be attributed to sample selection, different methodologies, and tissue specificity.

Kanda et al. (2015) reported that downregulation of *BTG1* mRNA in gastric cancer was positively associated with shorter disease-specific and recurrence-free survival of the patients with gastric cancer as an independent prognostic factor. BTG1 expression was adversely linked to poor prognosis of patients with hepatocellular, thyroid, nasopharyngeal, esophageal, breast, or non-small cell lung cancer (Sun et al., 2014b; Sun et al., 2014c; Sun et al., 2014d; Lu et al., 2014; Sheng et al., 2014). According to the Kaplan-Meier plotter, we found that *BTG1* expression was negatively correlated with favorable prognosis of gastric, lung, or ovarian cancer patients, but not for breast cancer patients. The correlation between *BTG1* expression and prognosis of the cancer patients did not parallel with the alteration in *BTG1* expression in cancer tissues. Additionally, TCGA data showed the same results about the prognostic significance of *BTG1* expression to Kaplan-Meier plotter’s in gastric and breast cancers although Kaplan-Meier plotter is based on cDNA array and the TCGA experiment on RNA sequencing. The tissue specificity might account for the phenomenon of the relationship between *BTG1* expression and prognosis.

Reportedly, human papillomavirus E7 peptide (p11-20) induced the pro-apoptotic *BTG1* expression in mature dendritic cells from human peripheral blood monocytes (Bard et al., 2004). BTG1 has been discovered to regulate quiescence in naïve and memory T cells (Kim et al., 2022). Loss of BTG1 expression causes glucocorticoid resistance both by decimating glucocorticoid receptor (GR) expression and the recruitment of BTG1/PRMT1 complex to GR promoter (van Galen et al., 2010). In the present study, we found that *BTG1* mRNA expression was closely linked to the infiltration of immune cells, indicating the possible role of *BTG1* in immunotherapy in future. The relationship between *BTG1* expression and infiltrating immune cells in gastric, breast and ovarian cancer was opposite to that in lung cancer, which might be attributable to its inclusion of lung adenocarcinoma and squamous cell carcinoma.

Because m6A-hypomethylation regulated FGFR4 phosphorylates GSK-3β and activates β-catenin/TCF4 signaling to drive anti-HER2 resistance, FGFR4 inhibition enhances susceptibility to anti-HER2 therapy in resistant breast cancer by reducing glutathione synthesis and Fe^2+^ efflux efficiency *via* the β-catenin/TCF4-SLC7A11/FPN1 axis and labile iron pool accumulation (Zou et al., 2022). Reportedly, TNBCs were classified into immune Phenotypes A and B according to the infiltration of stromal γδ T cells, CD4^+^ T cells, monocytes, M1 and M2 macrophages. In Phenotype A, there were rich immune-related pathways, and a stronger level of PD-L1, PD-1 and CTLA-4 (Zheng et al., 2020). Here, we found that *BTG1* mRNA expression was remarkably linked to the infiltration of immune cells, Her2 expression and molecular subtyping of breast cancer, demonstrating that *BTG1* would be employed to guide the target therapy for breast cancer patients.

KEGG analysis demonstrated that the *BTG1*-related pathways included cytokine-cytokine receptor interaction, cell adhesion molecules, chemokine, immune cell receptor and NF-κB signal pathways in gastric and breast cancers. The top hub genes mainly contained CD antigens in gastric cancer, FGF-FGFR and downstream genes in lung cancer, mitochondrial NADH: ubiquinone oxidoreductase in breast cancer, and ribosomal proteins in ovarian cancer respectively. These bioinformatics data provided novel clues to the molecular mechanisms of BTG1 in malignancies.

In conclusion, aberrant *BTG1* mRNA expression was closely linked to carcinogenesis, cancer aggressiveness, and worse prognosis of cancer patients in a tissue-specific manner. The study’s limitation is that the results from the Oncomine, TCGA, xiantao, UALCAN, and Kaplan-Meier plotter datasets were not validated using real-time RT-PCR, even after laser capture dissection because the specificity of cDNA chip or RNA sequencing is not enough high.

## Data Availability

The datasets presented in this study can be found in online repositories. The names of the repository/repositories and accession numbers can be found in the article/Supplementary Material.
